# Supplementary material for: Bromhexine Hydrochloride Prophylaxis of COVID-19 for Medical Personnel: A Randomized Open-Label Study
Source: Interdiscip Perspect Infect Dis. 2022 Jan 29;2022:4693121. doi: 10.1155/2022/4693121 (PMC8799951; doi:10.1155/2022/4693121)
Supplement: Supplementary Materials — Supplementary file contains study protocol translated to English. [file 4693121.f1.docx]

# Bromhexine Hydrochloride Prophylaxis of COVID-19 for Medical Personnel: A Randomized Open-Label Study

# by Mikhaylov EN, Lyubimtseva TA, Vakhrushev AD, Stepanov D, Lebedev DS, Vasilieva EYu, Konradi AO, Shlyakhto EV

**SUPPLEMENTS. Study protocol**

**_____________________________________________________________**

**Almazov National Medical Research Centre**

**Clinical study protocol**

**«Prophylaxis of SARS-CoV-2 infection and COVID-19 among medical personnel managing patients with the novel coronavirus disease »**

Contents

[1. Rationale 3](#_Toc73151362)

[2. Purpose 4](#_Toc73151363)

[3. Study type 4](#_Toc73151364)

[4. Inclusion criteria 4](#_Toc73151365)

[5. Exclusion criteria 4](#_Toc73151366)

[6. Drop out criteria 5](#_Toc73151367)

[7. Study design 5](#_Toc73151368)

[8. Study endpoints 6](#_Toc73151369)

[9. Randomization and sample size estimation 7](#_Toc73151370)

[10. Bromhexine hydrochloride dosing and administration rules 8](#_Toc73151371)

[11. Study timeline 8](#_Toc73151372)

[12. Subject exclusion 9](#_Toc73151373)

[13. Replacement of subjects when excluded from the study 9](#_Toc73151374)

[14. Preliminary study termination 9](#_Toc73151375)

[15. Safety reports 9](#_Toc73151376)

[16. Ethics 9](#_Toc73151377)

[Investigators 11](#_Toc73151378)

# 1. Rationale

Several independent scientific laboratories have analyzed data on the mechanism of action of drugs that block the transmembrane protease (TMPRSS2), which is necessary for the activation of the S protein of the coronavirus when it binds to the ACE2 receptor on the surface of epithelial cells. Currently, two drugs, potential TMPRSS2 inhibitors, are of particular interest: Camostat mesylate and Bromhexine hydrochloride [D. Stepanov, P. Lierz; Frenxiv, April 23, 2020; Markus Hoffmann, Hannah Kleine-Weber, Simon Schroeder et al. Cell 181, 271-280 April 16, 2020; Kailas Sonawane Sagar S. Barale Maruti J. Dhanavade et al. ChemRxiv April 22, 2020].

These drugs are effective chemical TMPRSS2 blockers in vitro. According to the available data on severe acute respiratory syndrome caused by the SARS-CoV type coronavirus, the mechanism of entry into the cell of the new SARS-CoV-2 coronavirus is similar: it has been demonstrated in animal models that the introduction of SARS-CoV-2 into epithelial cells can be blocked by Camostat mesylate. In mice, Camostat, dosed at concentrations similar to clinically achievable concentrations in humans, reduced mortality after infection with SARS-CoV from 100% to 30-35% [Yanchen Zhou, Punitha Vedantham, Kai Lu et al. Protease inhibitors targeting coronavirus and filovirus entry. Antiviral Research 116 (2015) 76-84; Hoffmann M, Kleine-Weber H, Schroeder S et al. SARS-CoV-2 Cell Entry Depends on ACE2 and TMPRSS2 and Is Blocked by a Clinically Proven Protease Inhibitor. Cell. 2020; 181: 1-10].

Bromhexine, a well known pharmacological drug, is an alkaloid derived from Adhatoda vasca used as a secretolytic expectorant for the treatment of respiratory diseases with excessive or viscous mucus. Bromhexine stimulates the activity of the ciliated epithelium, enhances lysosomal activity and causes hydrolytic depolymerization of mucous protein fibers [Bhagat A, Rachana R. Bromhexine: A Comprehensive Review. Int J Bio Med Res. 2018; 9 (3): 6455-9]. The concentration of Bromhexine in the pulmonary parenchyma two hours after administration is 2.4-5.9 times higher than in the blood plasma. The drug demonstrates first-order pharmacokinetics and is almost completely metabolized to a variety of hydroxylated metabolites; the most famous of these is Ambroxol, also available as a licensed mucolytic drug.

Like Camostat, Bromhexine inhibits the activity of TMPRSS2, thus preventing the SARS-CoV-2 virus from entering the cell. Bromhexine blocks the ability of TMPRSS2 to activate the tissue zymogen precursor, plasminogen activator in vitro [Lucas JM, Heinlein C, Kim T, Hernandez SA, Malik MS, True LD et al. The Androgen-Regulated Protease TMPRSS2 Activates a Proteolytic Cascade Involving Components of the Tumor Microenvironment and Promotes Prostate Cancer Metastasis. Cancer Discovery. 2014; 4: 1310-25].

It is important to note that only minor side effects of Bromhexine have been described, such as nausea (a common effect), rash, bronchospasm, vomiting, diarrhea, and fever (noted as a rare effect). Severe complications, such as Stevens-Johnson syndrome, or toxic epidermal necrolysis, possibly associated with the metabolite Ambroxol, are casuistic (no more than 0.16 cases per million patients).

There are currently no results of prospective studies of the use of Bromhexine hydrochloride as a preventive measure. There is one known study planned in China (Evaluating the Efficacy and Safety of Bromhexine Hydrochloride Tablets Combined With Standard Treatment / Standard Treatment in Patients With Suspected and Mild Novel Coronavirus Pneumonia (COVID-19) clinicaltrials.gov: NCT04273763; sample of 60 patients, start date studies 02/16/2020 Two prospective placebo-controlled studies are also known to study the effect of Camostat on reducing the severity of the incidence of COVID19: The Effect of Camostat Mesylate on COVID-19 Infection in Ambulatory Patients: An Investigator-Initiated Randomized, Placebo-Controlled, Phase IIa Trial, clinicaltrials.gov: NCT04353284, sample of 114 patients, study start date 04/30/2020 (Yale University, USA); The Impact of Camostat Mesilate on COVID-19 Infection: An Investigator-initiated Randomized, Placebo-controlled, Phase IIa Trial; clinicaltrials.gov: NCT04321096, sample of 180 patients, study start date - 03/31/2020 (Dani me, 9 participating centers).

We suggest, that when SARS-CoV-2 has already entered the cells, the effect of Bromhexine hydrochloride therapy will be low or insufficient. About 20-30% of medical personnel are infected with SARS-Cov-2. At the same time, there are similar data on the potential effectiveness of Bromhexine hydrochloride in the prevention of a new infection in risk groups from several independent groups of authors. According to data from personal communication with specialists in Germany, there were no cases of infection among those who took the drug prophylactically [Department of Anesthesiology, Intensive Care, Pain Management and Palliative Care, Marienkrankenhaus, Soest, Germany]. It can be assumed that there is a preventive effect of taking Bromhexine among medical personnel to reduce the risk of infection and COVID 19 disease, despite the use of personal protective equipment.

# 2. Purpose

To evaluate the efficacy and safety of oral administration of Bromhexine hydrochloride for the prevention of SARS-CoV-2 infection and COVID-19 disease in medical personnel managing patients with COVID-19

# 3. Study type

Prospective randomized open-label study with a control group, which assesses the superiority of prophylactic therapy

# 4. Inclusion criteria

1. Age ≥18 years;

2. Negative test (PCR) for SARS-CoV-2 infection;

3. Absence of clinical manifestations of a respiratory infection;

4. Presumptive management of patients with laboratory and/or clinically confirmed COVID-19;

5. Signed informed consent to participate in the study.

# 5. Exclusion criteria

1. Intolerance to Bromhexine hydrochloride;

2. Work outside of contact with SARS-CoV-2 infection;

3. Pregnancy (positive laboratory test for pregnancy or ultrasound confirmation of pregnancy) and breastfeeding period;

4. Other circumstances that the researcher considers inappropriate to participate in this study.

5. Peptic ulcer or duodenal ulcer, incl. in anamnesis

6. Documented contact with a SARS-CoV-2 carrier or a COVID-19 patient within 14 days prior to randomization

# 6. Drop out criteria

1. Failure to comply with the rules for the use of personal protective equipment when working with SARS-CoV-2 infection (the rules for the use of PPE in accordance with the recommendations of the Ministry of Health of Russia and internal orders of the General Director of the V.A. research; control of the use of PPE - independent);

2. Any acute illness not associated with SARS-CoV-2 that occurred during the study period

# 7. Study design

This is single-center study. Location of the study center:

Almazov National Medical Research Centre; 2, Akkuratova str., Saint-Petersburg 197341, Russian Federation

Study design is present on Figure 1.

**Figure 1. Study design**

**
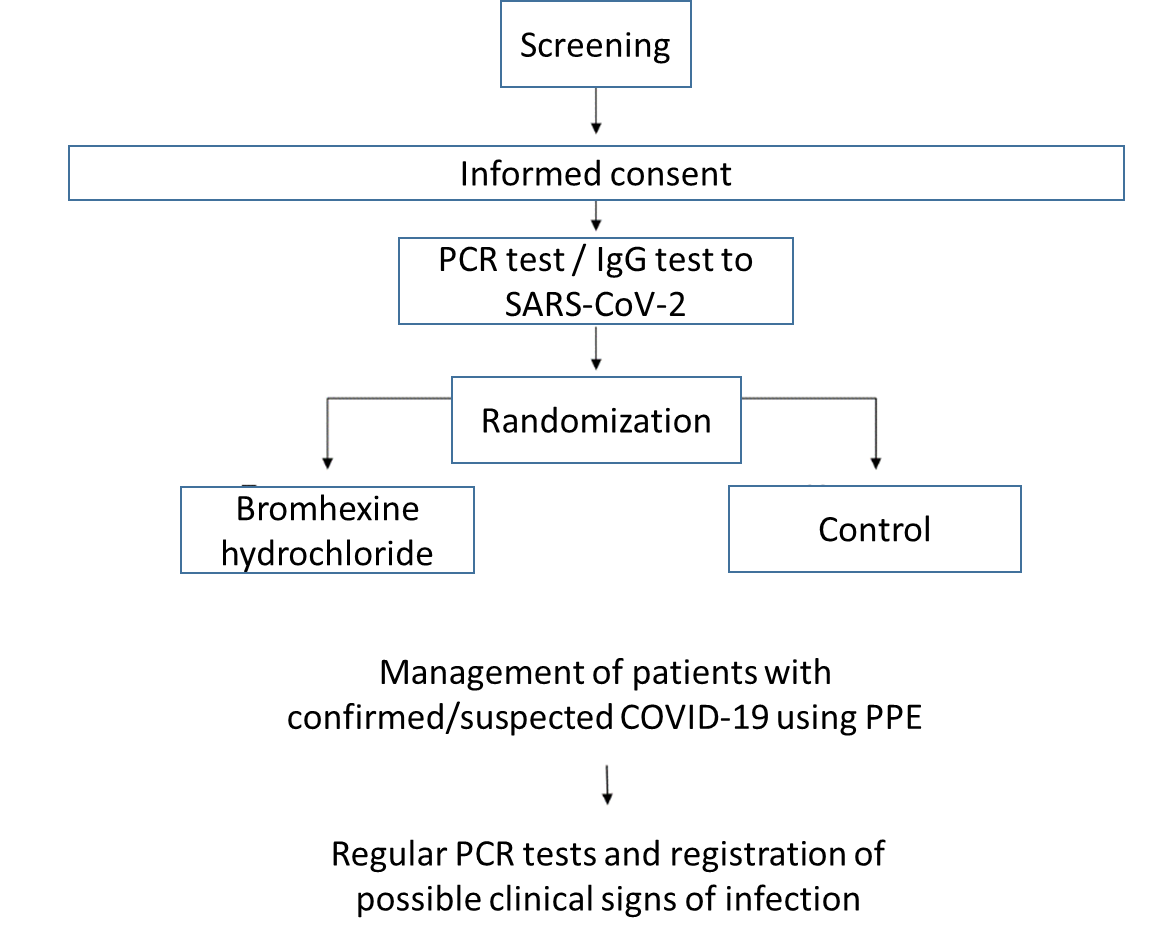
**

• When COVID-19 is expected clinically or SARS-CoV-2 infection is verified with a positive PCR test, the endpoint is reached and the drug may be canceled;

• However, the drug can be continued at the request of the participant for mild and moderate forms of the disease, since, theoretically, a decrease in viral load is possible; patients with continued therapy will continue to be followed, but outside the study protocol.

The same test systems for PCR (SARS-CoV-2 infection) will be used during the study.

# 8. Study endpoints

**8.1 Primary endpoint**

- Combined endpoint: a positive PCR swab test to SARS-CoV-a2 and/or clinically suspected COVID-19 at 4 weeks and/or after 14 days after the last contact with a SARS-CoV-2 positive person

**8.2 Secondary endpoints**

- The time to the onset of clinical symptoms of a respiratory infection with positive SARS-CoV-2 PCR from the moment of the first contact with COVID-19;
- Time to a positive swab PCR SARS-CoV-2;
- Number of symptomatic cases of SARS-CoV-2 infection in both study groups;
- The number of mild, moderate and severe forms of COVID-19;
- The presence and type of side effects of Bromhexine hydrochloride

**8.3 Methods for assessing primary and secondary endpoints**

1. Clinical assessment of study participants for the presence of symptoms of respiratory infection

2. Laboratory diagnostics of the presence of SARS-CoV-2: swab PCR test;

3. Chest computed tomography, pulse oximetry - to assess the severity of the disease;

4. Laboratory diagnostics of SARS-CoV-2 infection: plasma IgG;

5. Laboratory diagnostics of the severity of COVID-19: clinical blood test with leukocyte count, C-reactive protein, D-dimer, ALT, AST, bilirubin fraction; creatinine, urea, protein fractions; amylase; glucose;

6. The need for oxygen support and its type in the treatment of COVID-19; the duration of the need for oxygen therapy; duration of mechanical ventilation.

7. Working hours (hours) with patients with confirmed / suspected SARS-CoV-2 infection;

8. Additional observation of all study participants up to 8 weeks from the date of randomization.

**Methodology for calculating the time of work with SARS-CoV-2 infection**

1. Units of time calculation - hours;

2. Separately taken into account: a) the time of work in the therapeutic department, including the emergency room where patients with suspected / confirmed SARS-CoV-2 infection are located; b) the time of work in the intensive care unit, where patients with suspected / confirmed SARS-CoV-2 infection are located; c) the time of work in a "clean" area, outside the presence of patients with suspected / confirmed SARS-CoV-2 infection;

3. Working hours in the hospital without personal protective equipment in accordance with the regulations of Almazov National Medical Research Centre.

# 9. Randomization and sample size estimation

Randomization method: 1:1, allocation by minimization method to the preventive therapy group with Bromhexine hydrochloride and to the control group, taking into account the following factors: age (ranges 18-45, 45-64, 65-74, 75-79); type of contact with SARS-CoV-2 infection: ("red zone"; "green" zone); taking hydroxychloroquine to prevent COVID-19.

• "Red" zone: the closest and longest contact with SARS-CoV-2 infection associated with an infected aerosol (work in intensive care units and intensive care units; in operating and intervention laboratories; work with biological material of infected patients);

• Green Zone: work outside direct contact with patients with confirmed / suspected SARS-CoV-2 infection.

Known statistics:

• The incidence rate of medical personnel working with SARS-CoV-2 infection: ranges between 20 and 50% (https://www.cdc.gov/mmwr/volumes/69/wr/mm6915e6.htm?deliveryName=USCDC_425-DM25898);

• Frequency of serious side effects of bromhexine hydrochloride: no more than 0.16 cases per million patients (Ambroxol and bromhexine containing medicinal products. EMA / PRAC / 800767/2015, 10/09/2015);

Estimated effect of preventive therapy:

Empirically, it is suggested that prophylactic drug administration can reduce the incidence of the primary endpoint by 20%.

Calculation of the study sample size:

• Power: 80%

• Confidence level - alpha error: 5%

• The incidence of the primary endpoint in the control group is estimated 30%; the incidence of the primary endpoint in the study group is estimated 10%;

• Thus, the target sample size: 118 people (59 subjects in each group)

• Estimated dropout rate from study protocol (dropout after randomization, non-SARS-CoV-2 incidence) 15%; thus, the required number of participants in the study will be 139 people;

# 10. Bromhexine hydrochloride dosing and administration rules

The drug is administered orally, after meals, without chewing, with plenty of liquid:

3 times a day, 8 mg.

In case of impaired renal function (creatinine clearance <30 ml / min / 1.73 m2) and / or liver insufficiency (an increase in the level of hepatic transaminases by 2 or more times), the drug should be discontinued in this study.

# 11. Study timeline

**Table 1. Schedule of evaluations and prophylactic measures**

| Evaluations | 0 | 7 days | 14 days | 21 days | 28 days | 5-8 weeks |
| --- | --- | --- | --- | --- | --- | --- |
| Test (PCR) SARS-CoV-2 | + | + | + | + | + | + |
| Enzyme immunoassay - IgG to SARS-CoV-2 | +* |  |  |  |  |  |
| Clinical follow-up, daily | + | + | + | + | + | + |
| Bromhexine hydrochloride administration, daily (8 mg 3 times a day) | + | + | + | + | + | + |
| Individual protective equipment; daily | + | + | + | + | + | + |
| Concomitant pharmacological treatment record | + | + | + | + | + | + |
| Participant’s questionnaire maintaining | + | + | + | + | + | + |
| Laboratory diagnostics when SARS-CoV-2 is expected |  | When indicated | | | | |
| Chest computed tomography when SARS-CoV-2 infection is expected |  | When indicated | | | | |

*Plasma biobanking with subsequent IgG analysis when it is possible

# 12. Subject exclusion

Participants in the study can leave at any time, for any reason, without any consequences. A participant might be excluded from the study for a medical emergency.

**Study participant specific withdrawal criteria:**

• The presence of side effects of the use of Bromhexine hydrochloride, which complicates its further safe use and / or any other intolerance to the drug;

• The onset of pregnancy;

• Suspected / confirmed SARS-CoV-2 infection;

• Period of disability and / or hospitalization associated with SARS-CoV-2 infection.

# 13. Replacement of subjects when excluded from the study

Participants excluded from the study will not be replaced.

# 14. Preliminary study termination

The study may be terminated prematurely if there is a significant difference in the safety endpoints between groups after enrollment of 20 subjects, which indicates unacceptable safety. If the superiority of the treatment method is found early, there will be no reason for early termination of the study.

# 15. Safety reports

15.1 Adverse events (AE).

Adverse event is any unwanted event that may occur to a study subject during the study course, whether or not associated with bromhexine hydrochloride treatment. All adverse events reported by an investigator should be recorded.

Examples of adverse events based on studies in patients taking bromhexine:

Significant: allergic reaction in the form of anaphylactic shock;

Minor: nausea, vomiting, diarrhea, allergic reaction in the form of urticaria

15.2 Severe adverse events (SAE)

SAE – is any unwanted event, which in any from or duration may:
- cause death;

- is life-threatening (when it develops);

- requires unscheduled hospitalization or prolongs hospitalization period;

- leads to permanent or severe disability;

- any other important medical event that does not lead to death, is not life-threatening and does not require hospitalization, but may be interpreted (according to a specialist’s opinion) as an event with risk to a subject or needs an additional medical intervention for the prevention of an event.

When SAE is expected, an investigator must immediately inform the study coordinator and the primary investigator. The study coordinator and primary investigator are responsible for notification of the ethics committee.

# 16. Ethics

The study will be conducted in accordance with the principles of the Declaration of Helsinki (version 59 review of the WMA article, Seoul, Korea, October 2008).

15.1 Study enrollment and informed consent

All individuals who meet the inclusion and exclusion criteria are enrolled after they have given their consent. Information about the study will be provided by a physician-investigator, signed before the procedure begins, with the examination. The research participant will be given enough time to consider and decide on whether to participate or decline. The informed consent form has been reviewed and approved by the Ethics Committee of the Almazov National Medical Research Centre. Informed consent has been developed according to the ICH / GCP guidelines. Potential risks and participation in the study will be explained orally. A copy of the signed consent is issued to the research participant.

# Investigators

**Primary investigator**

Prof. Evgeny V. Shlyakhto, MD, PhD,

Director general, Almazov National Medical Research Centre

e.shlyakhto@almazovcentre.ru

**Executive committee**

Evgeny N. Mikhaylov, MD, PhD, Vice director, Institute of heart and vessels, Almazov National Medical Research Centre;

Prof. Alexandra O. Konradi, MD, PhD, vice-director, Almazov National Medical Research Centre;

Prof. Dmitry S. Lebedev, MD, PhD, head, arrhythmia department, Almazov National Medical Research Centre;

Tamara A. Lyubimtseva, MD, PhD, senior researcher, arrhythmia department, Almazov National Medical Research Centre

**Study coordinator**

Tamara A. Lyubimtseva, MD, PhD, senior researcher, arrhythmia department, Almazov National Medical Research Centre

**Co-investigators**

Dmitry Stepanov

Novikova, Anna N.

Vasilieva, Elena Yu.

Garkina, Svetlana V.

Orshanskaya, Viktoria S.

Vakhrushev, Aleksandr D.

Gasimova, Nigar Z.

Semenov, Andrey P.
